# Two miRNA prognostic signatures of head and neck squamous cell carcinoma: A bioinformatic analysis based on the TCGA dataset

**DOI:** 10.1002/cam4.2915

**Published:** 2020-02-17

**Authors:** Chaoying Wu, Lingxia Tong, Chaoqun Wu, Dong Chen, Jianguo Chen, Qianyun Li, Fang Jia, Zirui Huang

**Affiliations:** ^1^ Department of Otolaryngology Head and Neck Surgery The First Affiliated Hospital of Jinzhou Medical University Jinzhou Liaoning China; ^2^ Department of Ultrasound Jilin Cancer Hospital Changchun Jilin China; ^3^ Department of General Medicine Ningbo Medical Center Lihuili Hospital Ningbo Zhejiang China; ^4^ Jinzhou Medical University Jinzhou Liaoning China

**Keywords:** bioinformatics, head and neck cancer, microRNAs, survival

## Abstract

MicroRNAs(miRNAs) are maladjusted in multifarious malignant tumor and can be considered as both carcinogens and tumor‐inhibiting factor. In the present study, we analyzed the miRNAs expression profiles and clinical information of 481 patients with head and neck squamous cell carcinoma (HNSCC) through the TCGA dataset to identify the prognostic miRNAs signature. A total of 114 significantly differentially expressed miRNAs (SDEMs) were identified, consisting of 60 up‐adjusted and 54 down‐adjusted miRNAs. The Kaplan‐Meier survival method identified the prognostic function of 2 miRNAs (miR‐4652‐5p and miR‐99a‐3P). Univariate and multivariate Cox regression analyses indicated that the 2 miRNAs were significant prognostic elements of HNSCC. Furthermore, bioinformatic analysis was conducted by means of 4 online gene predicted toolkits to recognize the target genes, and enrichment analysis was performed on the target genes by DAVID. The outcomes depicted that target genes were correlated with calcium, as well as cell proliferation, circadian entrainment, EGFR, PI3K‐Akt‐mTOR, and P53 signaling pathways. Finally, the PPI network was conducted in view of STRING database and Cytoscape. Eight hub genes were identified by CytoHubba and MCODE app, respectively, CBL, SKP1, H2AFX, HGF, POLR2F, UBE2I, VAMP2, and GNAI2 genes. As a result, we identified 2 miRNAs signatures, 8 hub genes, and significant signaling pathways for estimating the prognosis of HNSCC. In order to further explore the molecular mechanism of HNSCC occurrence and development, more comprehensive basic and clinical studies are needed.

## Graphical abstract

This study provides us with a new insight that multiple miRNAs can be together used for predicting the prognosis of patients with HNSCC. In order to further confirm the prognostic value of the 2 miRNA signatures, our future research may focus on exploring the relationship between miRNA and genic profile of HNSCC.

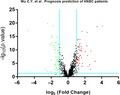

## 1 INTRODUCTION

Head and neck squamous cell carcinoma (HNSCC) is one of the sixth most common malignancies with about 600 000 new cases annually and more than 350 000 deaths worldwide.^1^ Current screening modalities are mainly limited to clinical test and imaging examinations, whose inaccuracy may lead to delays in detection. As a result, numerous patients with HNSCC thus suffered from terminal‐stage illness, leading to poor prognosis. The progress in improving survival rate of HNSCC is extremely tardiness.^2^ It is of great significance for early diagnosis and treatment of HNSCC to understand changes in molecular mechanisms and search for new biomarkers.^3^

MicroRNAs (miRNAs) are a group of evolutionary conserved small noncoding RNAs that exist in the form of RNA double transcripts of about 22 nucleotides.^4^ The miRNA lin‐4 in C. elegans was first discovered in 1993, and since then a large number of miRNAs have been detected in a variety of animals, plants, bacteria, and even viruses.^5^ MiRNAs are maladjusted in various malignant tumors, which can play the role of both oncogenes and tumor‐inhibiting factors.^6^ With the development of molecular biology and modern medical technology, miRNAs have been proved to affect numerous momentous physiological activities, including auxesis, multiplication, cell differentiation, apoptosis, cell cycle regulation, immune reaction, inflammation response, stress, and tumor invasion.^7^ Accordingly, the appraisal of miRNAs contributes to comprehend the biological mechanism of the pathema diagnosis, prognosis even to shed light on promoting personalized targeted therapies and precaution.^8^

Although a large number of studies have detected miRNAs and discussed its relationship with HNSCC survival and prognosis, due to the small sample size, biological heredity and mutation, and different experimental criteria, the repeatability of experimental results is sometimes questionable.^9^ A dependable survival prediction requires a large number of samples with detailed clinical characteristics. The Cancer Genome Atlas (TCGA), sponsored by National Cancer Institute (NCI), is a comprehensive open archive of cancer‐related molecular maps involving multidisciplinary and multi‐organization content.^10^ Today, TCGA data include more than 20 cancer types, providing some next‐generation sequencing (NGS), DNA sequencing, RNA expression, DNA methylation, microRNA sequencing, RNA sequencing (V1, V2), and clinically relevant meta data. All researchers have access to this raw and processed data.^11^ The molecular resources of TCGA are of great significance in our study of HNSCC.^12^

In our study, we analyzed miRNA expression profiles and clinical data of 481 HNSCC patients from the TCGA database to identify potential prognostic markers of HNSCC. The identified microRNAs can be regarded as a new biomarker for prognosis, which is helpful to further improve the clinical efficacy of HNSCC.

## 2 MATERIALS AND METHODS

### 2.1 Raw data of microarray refining and SDEMs ascertainment

The miRNAs expression data and clinical data of HNSCC were acquired from TCGA database through the UCSC Xena (https://xena.ucsc.edu/public). The inclusion criteria included the following: (a) the cases with miRNA expression and corresponding clinic data and (b) the cases with complete prognostic data. Ultimately, 481 cases were admitted in an actual project, 39 of which were detected by Illumina with adjacent normal tissue samples. The raw data of the clinical information and SDEMs are shown in Table S1. There are 39 HNSCC primary neoplasms that were matched with adjacent normal tissular samples for the SDEMs analysis in this study. The raw data of miRNAs were converted into a data matrix R. Afterwards, the data matrix were calculated via R package (Limma). The fold changes (FCs) of miRNAs data matrix were computed and SDEMs with |Log2(Fold change)| > 1 and cutoff criterion *P* < .05 were considered significant.

### 2.2 Correlation in SDEMs and the prognosis of patients with HNSCC

First, 481 patients corresponded to each SDEM were allocated into the living cluster, and deceased cluster on the basis of their survival status, and *T* test was performed according to SDEM expression value. Then, SDEMs with *P* < .05 were regarded as potential prognostic microRNAs, each prognostic miRNA was ranked, and each HNSCC patient was classified into high or low levels according to the median level represented. Besides, the approach of Kaplan‐Meier with log‐rank analysis was applied to figure out the role of the SDEMs for prognosis. Subsequently, a risk model was built through Cox proportional hazards regression model, risk score = $\sum_{i=1}^{n}\beta_{i}x_{i}$. Here, *β* was a symbol of partial regression coefficient, *x* is the expression value of SDEM. Finally, high‐ or low‐risk clusters were discerned via the rank of risk value, and the OS analysis was depicted by Kaplan‐Meier graph.

### 2.3 Ascertainment of desired genes related to prognostic SDEMs

Four cyber algorithms, TargetScan (http://www.targetscan.org v7.2 last updated 3/2018), DIANA‐microT‐CDS (http://www.microrna.gr/microT-CDS v5.0 last updated 7/2012), miRWalk (http://zmf.umm.uni-heidelberg.de/apps/zmf/mirwalk2/ last updated 2/2016), and miRTarbase (http://mirtarbase.mbc.nctu.edu.tw/php/index.php/ last updated 9/2017), were utilized to forecast the downstream desired gene of the related prognostic SDEMs. Furthermore, the Venn Diagram v2.1 and Funrich v3.1.3 were used to illustrate the predicted desired genes from all the toolkits to further determine the bioinformatics credibility.

### 2.4 Function analysis of target genes

DAVID is a dependable procedure used for enrichment analysis and display of biological functional annotations. The gene ontology (GO) terms, which consists of 3 main offtakes, is a significant online function annotation tool in genome research. The KEGG passway is a widely used biological databases and comprehensive database resource annotated through completely genomes sequencing. Overlapping genes predicted by at least 2 prediction platforms were selected as target genes. Thereafter, DAVID was used to conduct enrichment analysis of target genes, and the underlying mechanism of SDEMs HNSCC was identified in the context of GO and KEGG pathways. *P* value <.05 and gene count ≥2.0 were the critical values.

### 2.5 PPI network analysis

After functional enrichment analyses of SDEGs, a protein‐protein interaction network was carried out on account of STRING and Cytoscape software. Based on the STRING database, the target gene was selected with the score (highest confidence) >0.9, and interaction network was then showed through Cytoscape software. Molecular Complex Detection (MCODE) app was utilized for analyzing the entire network. Besides, hub genes were recognized by the CytoHubba app. As an important node of gene interaction in PPI network, the hub genes are a small number of genes with many interaction partners. According to 11 classification methods in cytoHubba, hub genes were identified by overlapping the first 10 genes.

## 3 RESULTS

### 3.1 Ascertainment of HNSCC SDEMs

**Figure 1:**
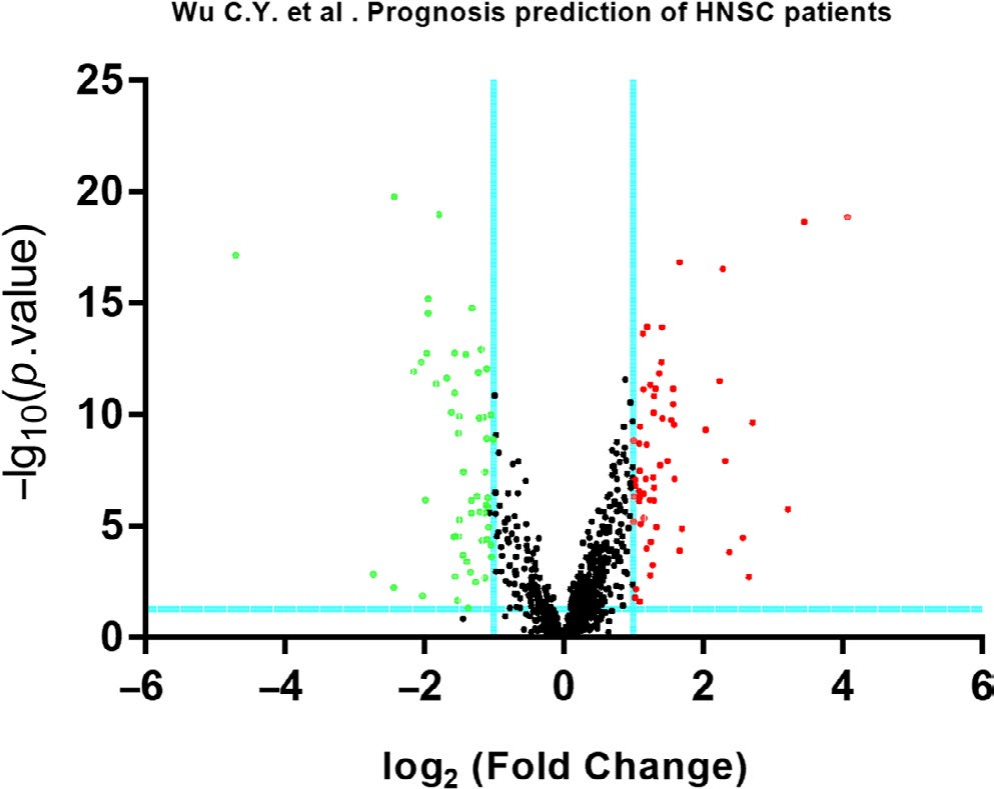
The volcano plot of the significantly differentially expressed miRNAs. A total of 114 SDEMs were identified between 39 pairs of HNSCC patients and adjacent normal tissues (under the conditions of *P* < .05 and |log2FC|> 1.0). Green represents down‐adjusted miRNAs and red represents up‐adjusted miRNAs

**Figure 2:**
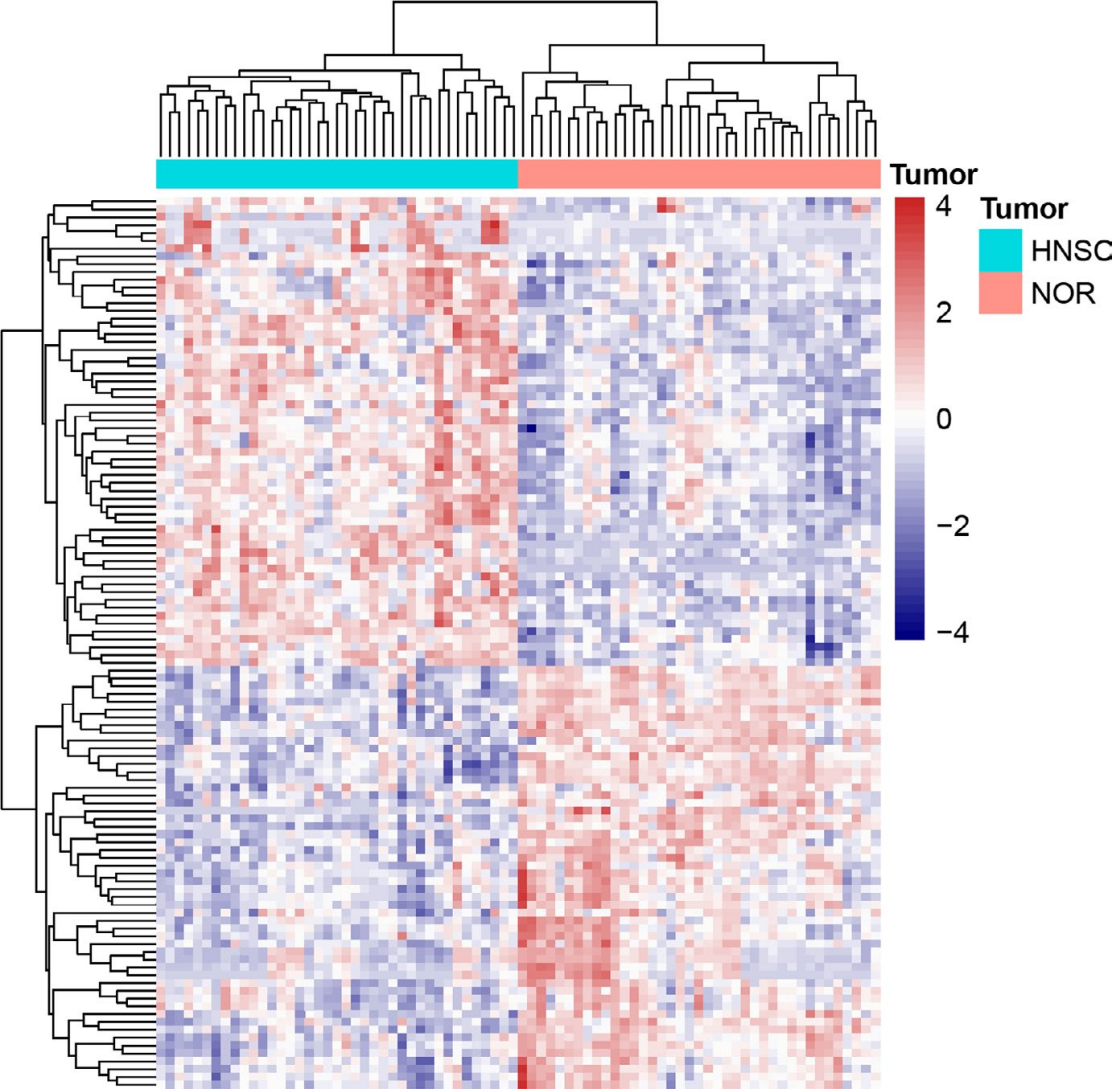
Hierarchical clustering analysis of the SDEMs between 39 pairs of HNSCC and adjacent normal sample. Each row represents the expression level of a miRNA, and each column represents a sample. The pink color in the heatmaps indicates Head and Neck squamous cell carcinoma samples, the blue color indicates normal samples

The raw Illumina platform dataset, including 39 HNSCC primary tumor matched with 39 adjacent normal tissue samples, were procured through TCGA omnibus. In accord with the cutoff standard, 114 SDEMs were recognized in HNSCC primary tumor tissues and adjacent normal tissue samples, including 60 up‐adjusted and 54 down‐adjusted miRNAs. (Table S2). The outcomes of 60 up‐adjusted and 54 down‐adjusted miRNAs were depicted in scatter diagram of the Volcano (Figure 1). The visualization of hierarchical cluster analyze revealed the distinction about SDEMs between HNSCC primary tumor and adjacent normal tissue samples (Figure 2).

### 3.2 Appraisal of SDEMs associated with overall survival rate in HNSCC

**Figure 3:**
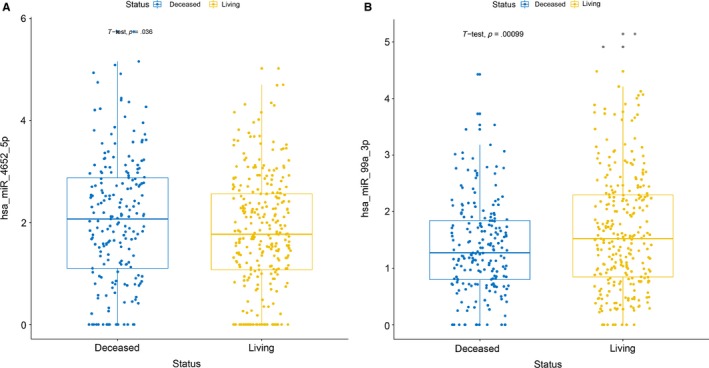
The Student's *T*‐test of the two DEMs expression value between Living and Deceased group. The results were statistical difference (A:miR‐4652‐5p: *P* = .036; B:miR‐99a‐3p: *P* = .00099)

We acquired 481 cases corresponded to each SDEM in order to determine the role of the SDEMs in predicting the OS of HNSCC patients. All clinical data including age of initial diagnosis, TNM stage, histological grade, gender, and clinical stage are shown in Table S3. The *T* test of the 2 SDEMs expression value between living and deceased group was carried out and the results were statistically significant (miR‐99a‐3p: *P* = .00099, miR‐4652‐5p: *P* = .036). The visualization is shown in Figure 3. The curves of Kaplan‐Meier method and logarithmic‐rank test displayed that miR‐99a‐3p was a positive factor for OS; on the contrary, miR‐4652‐5p was a negative one (Figure 3). Correlation analysis of 2 kinds of SDEMs with clinical information displayed that miR‐99a‐3p was significantly correlated with histologic grade (*P* = .028), T stage (*P* = .02), and N stage (*P* = .05) and miR‐4652‐5p was related to M stage (*P* = .03) and T stage (*P* = .046) (Table 1).

**Table 1:** Association of 2 differentially expressed miRNAs and clinical features

Variables	miR‐4652‐5p expression		*P* value	miR‐99a‐3p expression		*P* value
	Low	High		Low	High	
Age at diagnosis						
<60	113	101	.514	106	108	.887
≥60	133	134		134	133	
Gender						
Male	173	177	.219	170	180	.342
Female	73	58		70	61	
T stage						
T1 + T2	97	74	.046	73	98	.02
T3 + T4	139	156		159	136	
N stage						
N0	112	110	.862	100	122	.05
N1‐3	122	116		130	108	
M stage						
M0	230	222	.03	225	227	.996
M1	0	5		3	2	
Histologic grade						
G1 + G2	172	167	.988	181	158	.028
G3 + G4	62	60		51	71	
Clinical stage						
I + II	59	49	.371	48	60	.206
III + IV	179	181		185	175	

### 3.3 Prognostic effects of 2 SDEM features

We built a risk model with a Cox proportional risk regression model and calculated the risk score of 481 HNSCC cases. Then, we sorted 481 cases by the median risk score.

**Figure 4:**
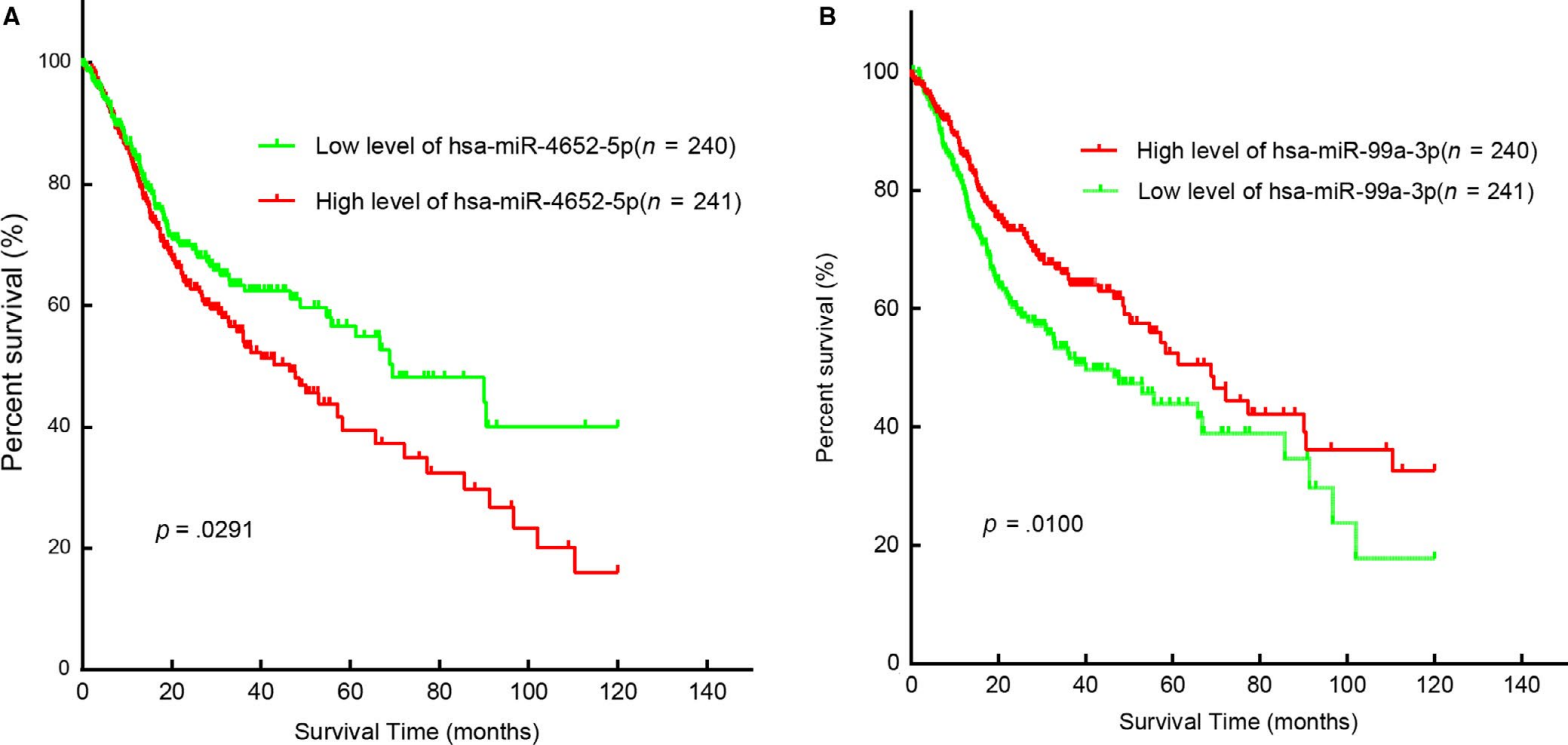
Two differentially expressed miRNAs were associated with overall survival in Head and Neck squamous cell carcinoma by using Kaplan‐ Meier curve and Log‐rank.(A:miR‐4652‐5p; B:miR‐99a‐3p)

Consequently, all 481 cases were divided into high‐risk group or low‐risk group and the Kaplan‐Meier method with log‐rank test was performed. The outcome of the OS between these 2 groups was statistically and remarkably different (*P* = .0031). (Figure 4). Moreover, multivariate and univariate Cox regression analyses were used to identify the prognostic impact of 2 SDEMs features in accordance with the correlation between 2 SDEMs and clinical elements. The result showed that the age (HR [95% CI] = 1.385, *P* = .033), the 2 SDEMs (HR [95% CI] = 1.464, *P* = .01), and T stage (HR [95% CI] = 1.430, *P* = .029) were statistically and remarkably related to prognostic status in HNSCC patients (Cox regression univariate analysis). Besides, the age (HR [95% CI] = 1.372, *P* = .038), T stage (HR [95% CI] = 1.386, *P* = .047), and the 2 SDEMs (HR [95% CI] = 1.431, *P* = .016) according to Cox regression multivariate analysis were independent prognostic factors of HNSCC patients (Table 2).

**Table 2:** Univariate and multivariate Cox regression analyses in HNSCC patients

Variables	Univariate analysis		Multivariate analysis	
	HR (95%CI)	*P* value	HR (95%CI)	*P* value
Age at initial diagnosis (≥60 vs < 60)	1.385 (1.027‐1.869)	.033	1.372 (1.017‐1.851)	.038
Gender (Female vs Male)	1.317 (0.967‐1.794)	.081		
T stage (T3 + T4 vs T1 + T2)	1.430 (1.037‐1.972)	.029	1.386 (1.004‐1.912)	.047
N stage (N1‐3 vs N0)	1.097 (0.823‐1.462)	.528		
M stage (M1 vs M0)	2.374 (0.757‐7.448)	.138		
Clinical stage (III + IV vs I + II)	1.234 (0.864‐1.761)	.247		
Histologic grade (G3 + G4 vs G1 + G2)	0.850 (0.613‐1.179)	.33		
SDEM‐signature (High risk vs Low risk)	1.464 (1.094‐1.959)	.01	1.431 (1.068‐1.917)	.016

### 3.4 Prediction of target genes of 2 SDEMs and analysis of GO and KEGG

Predicted genes of miR‐4652‐5p and miR‐99a‐3P could be obtained by target prediction instruments: TargetScan, miRTarbase, DIANA‐microT‐CDS, and miRWalk.

**Figure 5:**
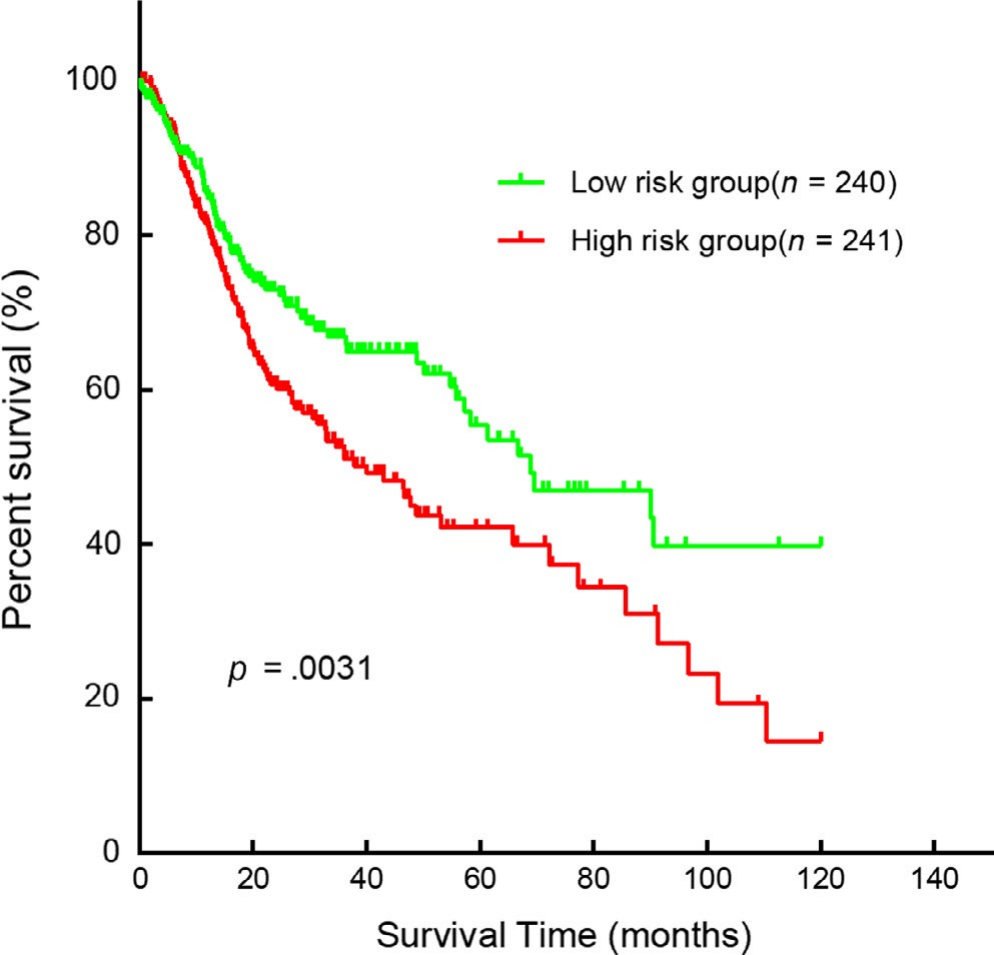
Kaplan‐Meier curve for the two‐miRNA signature in HNSCC. The patients were categorized into high‐risk group and low‐risk group based on median Risk‐Score. Compared to the low‐risk group, patients in the high‐risk group had a poorer prognosis (*P* = .0031)

**Figure 6:**
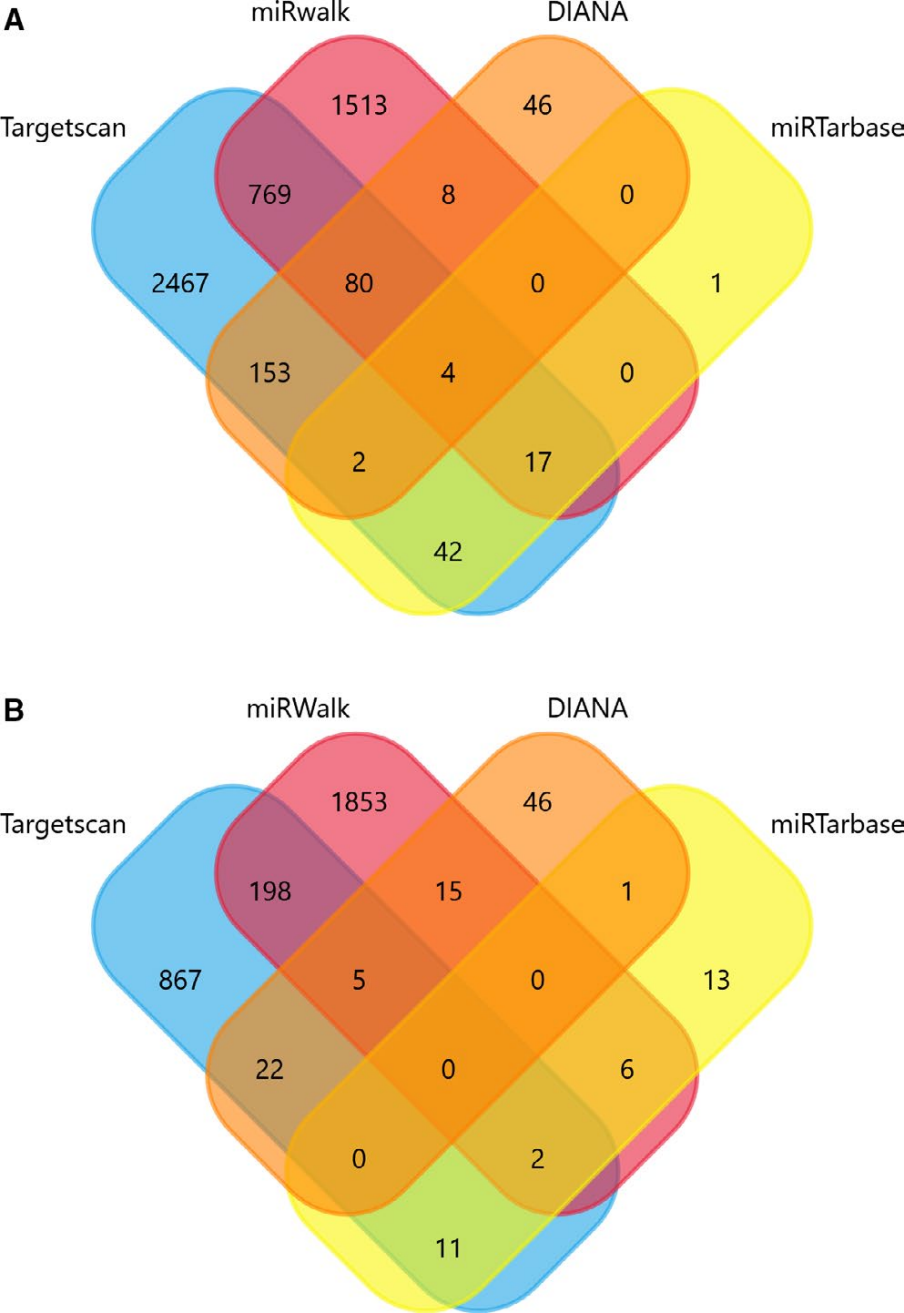
Venn diagram depicting the number of predicted target genes from miRanda, miRDB, TargetScan, and DIANA‐microT‐CDS. The overlaps indicated the numbers of genes predicted by more than one algorithm. (A: miR‐4652‐5p; B: miR‐99a‐3P)

By selecting at least 2 predicted genes from each SDEM as target genes (Figure 5), we identified 1335 target genes. GO analysis was then performed to determine the biological processes, cellular components, and molecular function enriched in target genes set (Figure 6). The BP included multicellular organism growth, synaptic vesicle exocytosis, cell proliferation, and EGFR. MF indicated actin filament binding and protein binding as important related processes. CC showed that postsynaptic density, Golgi membrane, and membrane are important components. Moreover, KEGG pathways enriched in calcium signaling pathway, circadian entrainment, dopaminergic synapse, and P53 signaling pathways, which suggested that target genes may be associated with cancer.

### 3.5 PPI network construction and analysis of modules

**Figure 7:**
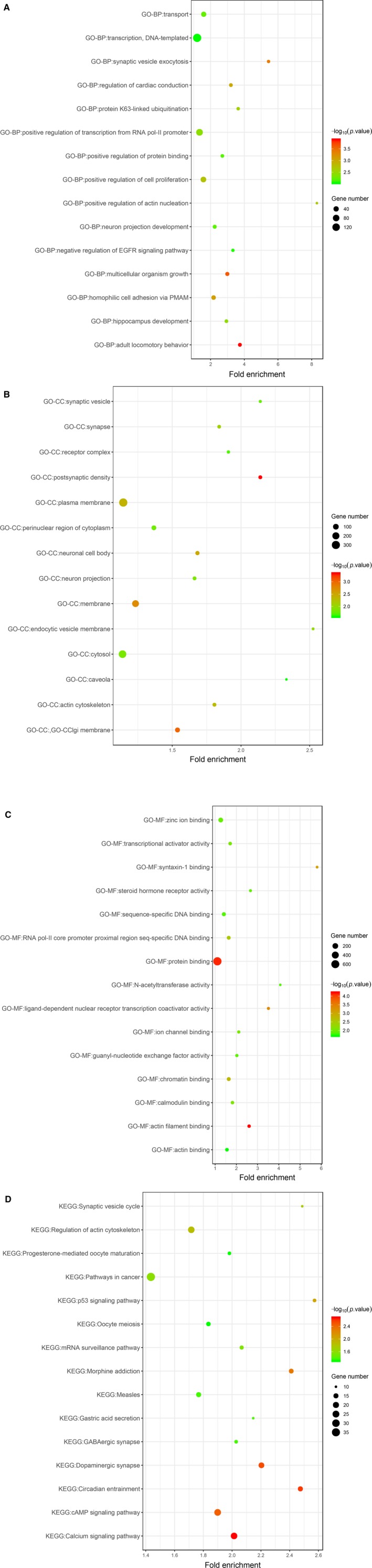
The functional enrichment analysis showed the significant enriched GO biological processes and KEGG pathway of target genes (A:BP; B:CC; C:MF; D:KEGG)

**Figure 8:**
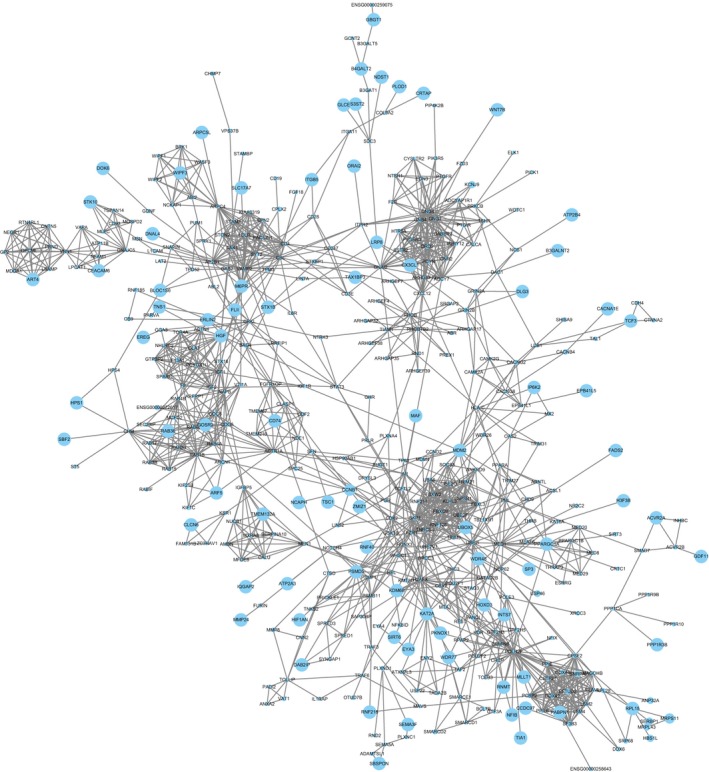
The STRING database was used to mine the PPI pairs of differentially expressed genes. We identified 1579 PPI pairs among 1335 SDEGs and established a PPI network. The size of the degree is displayed according to the size of the node. The larger degree value represented the larger the node diameter

**Figure 9:**
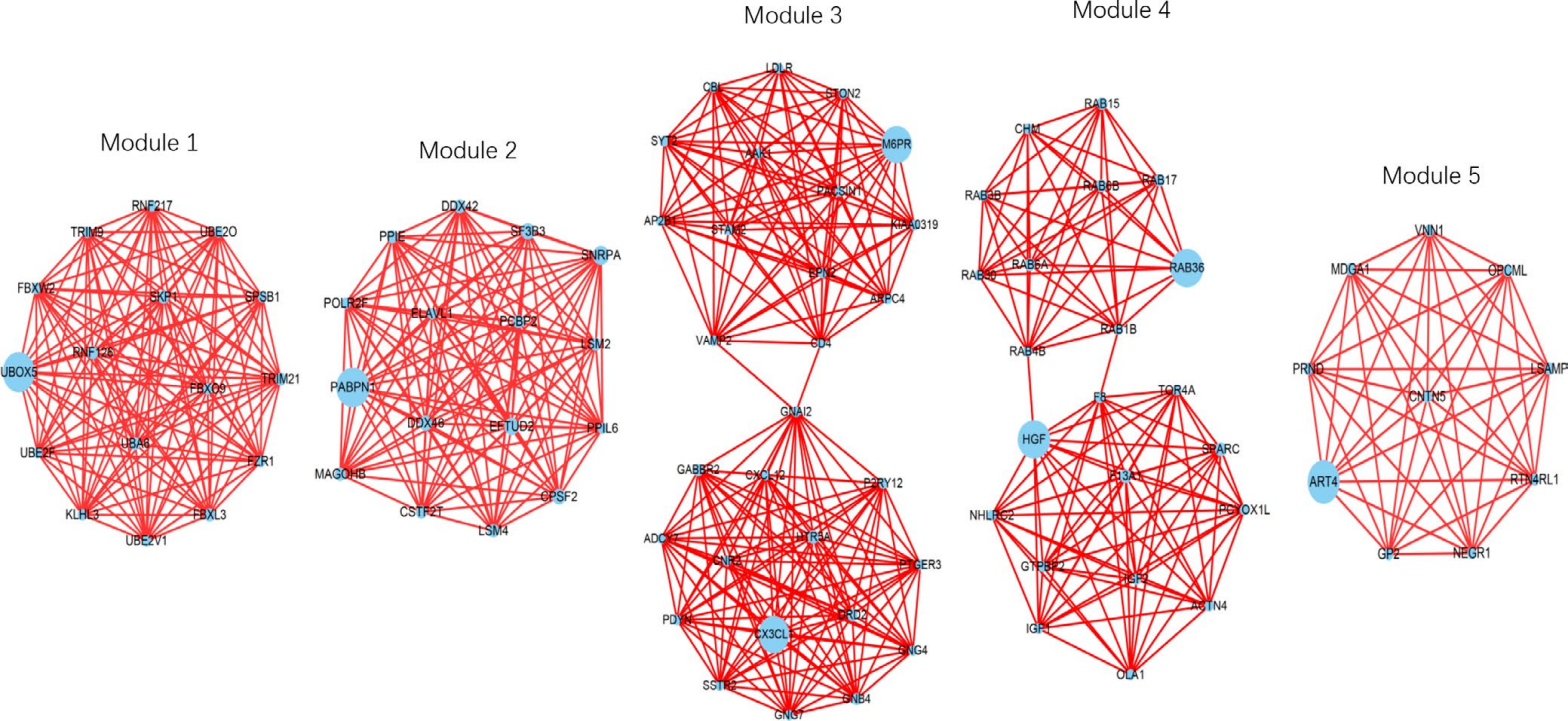
The significant modules in HNSCC. Each module was extracted after mapping SDEGs to the entire PPI network using the Cytoscape MCODE plugin. MCODE scores >10 and a number of nodes >10 were set as cut‐off criteria with the default parameters (Degree cutoff ≥2, Node score cut‐off ≥0.2, K‐core ≥2 and Max depth = 100). Node size is shown according to its network degree

**Figure 10:**
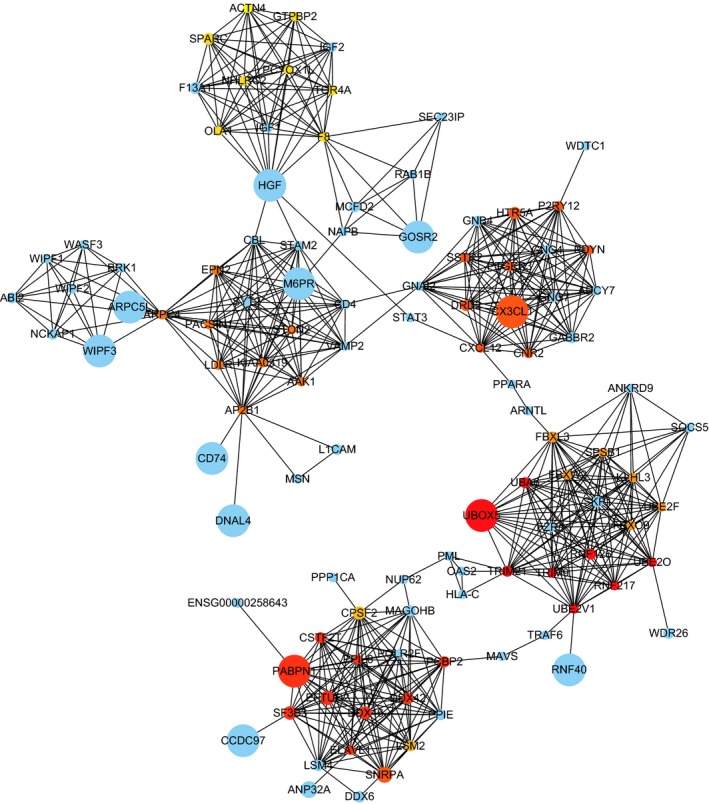
The DMNC algorithm of CytoHubba in the PPI network. Top 10 hub genes with a higher degree of connectivity was identified, including RNF217, RNF126, UBA6, UBE2V1, UBE2O, TRIM21, UBOX5, TRIM9, EFTUD2, SF3B3

In the entire PPI network (Figure 7), using the Molecular Complex Detection (MCODE) app, 5 major modules were extracted as shown in Figure 8. The enrichment analysis of these subnetworks also corroborated the effectiveness, and they were found to relate to Spliceosome, GABAergic synapse, morphine addiction, chemokine signaling pathway, pathways in cancer, ubiquitin‐mediated proteolysis, circadian entrainment, and alcoholism (Table 3). Combining the results of 11 ranked methods of cytoHubba, a total of 8 hub genes, such as CBL, SKP1, H2AFX, HGF, POLR2F, UBE2I, VAMP2, and GNAI2, were selected (Table S4, Figure 9). The hub genes identified by DMNC ranked methods were is shown in Figure 10. To further verify the gene could be used in further biomarker identification, we evaluated our result in another public casuistry of HNSCC. Based on the GEO2R tool, we screened DEMs between the patients with HNSCC and normal individuals. The result was validated in external cohorts from the NCI (National Cancer Institute) cohort (GSE10751) (Table S5).

**Table 3:** Pathway enrichment analysis of differentially expressed genes in the 5 modules based on information from the Kyoto Encyclopedia of Genes and Genomes (KEGG) pathways database

KEGG pathway	*P* value
Spliceosome	5.32E‐06
GABAergic synapse	5.11E‐04
Morphine addiction	6.61E‐04
Chemokine signaling pathway	.001767141
Pathways in cancer	.002621785
Ubiquitin‐mediated proteolysis	.003992899
Circadian entrainment	.006173793
Alcoholism	.007252256
Retrograde endocannabinoid signaling	.007635173
Serotonergic synapse	.010228883
Cholinergic synapse	.010548241
Glutamatergic synapse	.010874016
cAMP signaling pathway	.015890647
Dopaminergic synapse	.016561176
Progesterone‐mediated oocyte maturation	.028192343

## 4 DISCUSSION

In the last few decades, with an expeditious comprehension of the molecular biology of HNSCC as well as conservative treatment of radiochemotherapy and radical surgery over the last few decades, the morbidity and mortality of HNSCC have decreased to a certain extent.^13^

Nevertheless, there are almost 650 000 new patients appear worldwide each year, and half of them die after undergoing various treatments such as radical surgery, radiotherapy, chemotherapy, and targeted therapy.^14^ The prognosis of HNSCC patients is not optimistic, and the 5‐year overall survival rate is still disillusionary, which forms a sharp contrast to other primary cancers.^9^ Therefore, a complete and systematic cognition of the molecular mechanisms of HNSCC and the identification of novel biomarkers may provide novel therapeutic strategies for ameliorating to ameliorate the prognosis of patients with HNSCC.^15^

For decades, multiple researchers had reported that miRNAs could possess oncogenic function or play a tumor suppressing role in mediating cellular biological behavior. Abundant evidence had verified that miRNAs construct a complex network of genic expression and biologic pathway regulation, which has the function of prognosis and treatment target in many kinds of cancers.^16^ So far, several studies had proofed certain miRNAs with prognostic roles, such as miR‐654‐5p,^17^ miR‐125b,^18^ microRNA‐194‐5p,^18^ miR‐196b,^19^ miR‐548b,^20^ and so on. Nevertheless, the majority of formerly researches have concentrated on unimolecular incidents, limited cases, nonstandardized detective technique, and so on. We collected and analyzed the data of HNSCC patients in the public database. Hui et al published that miR‐1251, miR‐618, and miR‐328 were potentially significant prognostic markers of HNSCC. According to Wong et al, miR‐193b‐3p, miR‐455‐5p, miR‐92a‐3p, and miR‐497‐5p were associated with survival in OPSCC. In our research, we identified 114 SDEMS, including 60 up‐regulated SDEMS and 54 down‐regulated SDEMS, of which 2 (miR‐4652‐5p, miR‐99a‐3P) were associated with survival in patients with HNSCC. The difference of the selected samples is the reason of identifying miRNAs with different prognosis.

After that, we parallelled and detected our findings with current publications. According to Yury et al, miR‐4652 was up‐adjusted in HNSCC, which was suggested to function as a carcinogen and proved to be involved in the initiation and adjustment of significant cancer pathogenesis.^14^ Hu et al suggested the miR‐4652 high potential for involvement in cancer development.^21^ Kuo et al declared that miR‐99a‐5p could repressed the intrusion and metastasis of oral cancer cells partly by reducing myotubularin‐related protein 3 (MTMR3) expression.^22^ Wong and colleagues reported that the decrease in miR‐99a expression was found in tongue squamous cell carcinomas, suggesting that miR‐99a has an anticancer effect.^23^ Chen et al indicated that by targeting IGF1R and mTOR signaling pathways miR‐99a/b were inhibited after allotopic transfection and miR‐99a was involved in tumorigenesis.^24^ Chen and Hu et al analyzed that the expression of miR‐99a decreased remarkably in OSCC and miR‐99a took part in the early detection and prognosis of OSCC.^25^ MiR‐99a‐5p affected the vitality and proliferation, migration together with the invasion of oral cancer cells through targeting NOX4.^26^ Therefore, we further screened the target genes of 2 miRNA signatures and predicted the GO function and KEGG pathway of target genes through bioinformatic approaches.

Dysregulation of genic expression could have an impact on tumorigenesis and evolvement through the maladjusted molecular mechanism and signaling pathways. Furthermore, in our study, we performed 4 internet target prediction toolkits to recognize the objective genes of 2 miRNA signatures. Then, the target genes were enriched and analyzed by DAVID, and the outcomes depicted that these objective genes were correlated with circadian entrainment, EGFR, calcium, cell proliferation, and P53 signaling pathways. Zhao's team indicated that calcium signaling pathway was reduced and played a crucial role in regulating the start‐up and development of OSCC. In addition, deregulation of the calcium signal was involved in tumor initiation, angiogenesis, progression, and metastasis.^27^ Tang et al suggested that the circadian clock gene restrained the growth of tumors and enhanced the sensitivity of Taxol to TSCC, as a result of which, circadian entrainment regulated cancer development and chemotherapy susceptibility.^28^ Georgios et al reported that in human laryngeal cancer, acting‐binding protein regulates the replacement of acting filaments, and the focal agglutination signal of acting cytoskeleton is closely related to tumor invasion, metastasis, and cell migration.^29^ Dionysopoulos et al published that EGFR activation was mediated by its downstream signaling pathway PI3K‐Akt‐mTOR in laryngeal squamous cell carcinoma, in which the high expression of mTOR signaling pathway was correlated with poor prognosis.^30^ Osman et al supported that p53 signaling pathway acted as a predicting apparatus of prognosis, which decided whether the laryngeal function can be preserved in an advanced stage of head and neck squamous cell carcinoma.^31^ Therefore, the results of functional enrichment analysis are consistent with our study.

Meanwhile, 8 hub genes, CBL, SKP1, H2AFX, HGF, POLR2F, UBE2I, VAMP2, and GNAI2, were chosen according to the HNSCC PPI network. A section of these genes has been reported as biomarkers in previous studies. Cleo et al reported that the low expression level of c‐CBL acted as a tumor suppressor and may be a potential therapeutic target in patients with HNSCC.^32^ Natalie et al demonstrated that HGF/c‐Met signaling pathway, as a mechanism of resistance against EGFR inhibition, was provided with carcinogenic effect, thus the HGF/c‐Met signaling pathway was investigated as a novel therapeutic target in HNSCC.^33^ Hu et al indicated that the expression of SKP1 was decreased in HPSCC, which showed the significance of SKP1 in diagnostic and prognostic tests.^34^ Lu et al identified that GNAI2, as an oncogene, may be involved in TSCC start‐up, occurrence, and development.^35^ In esophageal squamous cell carcinoma (SCC), UBE21‐v5 is regarded as a carcinogenic factor by inhibiting the activity of CIEBPa and/or p53.^36^ On the whole, these hub genes could exert considerable influence on the development of HNSCC.

In conclusion, we identified 2 miRNA signatures, 8 hub gene and significant signaling pathways for estimating the prognosis of patients with HNSCC. Furthermore, more comprehensive basic and clinical studies are necessary to explore the occurrence and progress of further molecular functions in HNSCC.

## CONFLICT OF INTEREST

The authors declare that they have no competing interests.

## AUTHORS’ CONTRIBUTIONS

Chaoying Wu conceptualized this work, provided study's design, coordination of the study, and wrote the initial and final version of the manuscript. Chaoying Wu and Chaoqun Wu downloaded data from databases, managed and analyzed clinical data, and performed microarray data analysis, including data quality control. Qianyun Li, Zirui Huang, and Fang Jia performed survival prediction and statistical and gene network analyses. Dong Chen, Jianguo Chen, and Lingxia Tong analyzed biological results. All the authors have read and approved the final manuscript.

## Data Availability

The data used to support the findings of this study are available from the corresponding author upon request.
