# Supplementary material for: Two miRNA prognostic signatures of head and neck squamous cell carcinoma: A bioinformatic analysis based on the TCGA dataset
Source: Cancer Med. 2020 Feb 17;9(8):2631–42. doi: 10.1002/cam4.2915 (PMC7163094; doi:10.1002/cam4.2915)
Supplement: Supplementary file 2 [file CAM4-9-2631-s002.doc]

**Table S2.** Differentially expressed miRNAs between HNSC tissues and normal tissues

Up miRNAs Down miRNAs

| miRNAs | logFC | *P* Value | FDR | miRNAs | logFC | *P* Value | FDR |
| --- | --- | --- | --- | --- | --- | --- | --- |
| hsa-miR-744-3p | 1.009708 | 6.44E-06 | 7.56E-05 | hsa-miR-375 | -4.6938 | 6.97E-18 | 2.34E-15 |
| has-miR-301a-5p | 1.010724 | 1.44E-09 | 4.39E-08 | hsa-miR-1-3p | -2.72059 | 0.001454 | 0.008474 |
| hsa-miR-450a-5p | 1.011366 | 9.15E-08 | 1.74E-06 | hsa-miR-133a-3p | -2.42873 | 0.005643 | 0.026976 |
| hsa-miR-338-5p | 1.016657 | 4.85E-07 | 7.88E-06 | hsa-miR-99a-5p | -2.42211 | 1.68E-20 | 2.82E-17 |
| hsa-miR-3660 | 1.035025 | 1.50E-07 | 2.77E-06 | hsa-miR-30a-3p | -2.1419 | 1.19E-12 | 9.49E-11 |
| hsa-miR-940 | 1.036555 | 8.44E-08 | 1.63E-06 | hsa-miR-29c-3p | -2.0356 | 4.54E-13 | 4.01E-11 |
| hsa-miR-34c-3p | 1.043787 | 0.006693 | 0.030854 | hsa-miR-6510-3p | -1.97867 | 6.71E-07 | 1.03E-05 |
| hsa-miR-24-2-5p | 1.08908 | 2.69E-07 | 4.76E-06 | hsa-miR-204-5p | -1.95414 | 1.86E-13 | 1.95E-11 |
| hsa-miR-93-3p | 1.089446 | 2.03E-09 | 5.98E-08 | hsa-miR-139-3p | -1.93797 | 2.83E-15 | 4.75E-13 |
| hsa-miR-17-5p | 1.091166 | 7.71E-07 | 1.14E-05 | hsa-let-7c-5p | -1.93517 | 6.19E-16 | 1.30E-13 |
| hsa-miR-452-3p | 1.096797 | 3.19E-08 | 7.05E-07 | hsa-miR-30a-5p | -1.82225 | 4.13E-12 | 2.57E-10 |
| hsa-miR-96-5p | 1.098411 | 5.23E-07 | 8.36E-06 | hsa-miR-101-3p | -1.78141 | 1.06E-19 | 7.51E-17 |
| hsa-miR-4746-5p | 1.105122 | 3.34E-10 | 1.17E-08 | hsa-miR-195-5p | -1.66912 | 2.28E-12 | 1.60E-10 |
| hsa-miR-182-5p | 1.108425 | 8.08E-06 | 9.29E-05 | hsa-miR-139-5p | -1.60511 | 8.12E-11 | 3.67E-09 |
| hsa-miR-671-5p | 1.14121 | 2.36E-14 | 3.05E-12 | hsa-miR-486-5p | -1.56951 | 3.06E-05 | 0.000296 |
| hsa-miR-130b-5p | 1.149437 | 7.21E-12 | 3.90E-10 | hsa-miR-100-5p | -1.55696 | 1.77E-13 | 1.95E-11 |
| hsa-miR-424-5p | 1.15208 | 3.60E-07 | 6.03E-06 | hsa-miR-136-3p | -1.55689 | 1.09E-11 | 5.72E-10 |
| hsa-miR-21-3p | 1.155026 | 4.37E-06 | 5.35E-05 | hsa-miR-144-3p | -1.55166 | 2.79E-05 | 0.000275 |
| hsa-miR-135b-5p | 1.159017 | 4.61E-06 | 5.60E-05 | hsa-miR-208b-3p | -1.54907 | 0.001788 | 0.010069 |
| hsa-miR-4326 | 1.184783 | 7.55E-08 | 1.49E-06 | hsa-miR-381-3p | -1.50656 | 2.99E-05 | 0.000292 |
| hsa-miR-615-3p | 1.194387 | 0.000101 | 0.000827 | hsa-miR-125b-2-3p | -1.50527 | 6.97E-10 | 2.29E-08 |
| hsa-miR-301a-3p | 1.196536 | 2.21E-09 | 6.40E-08 | hsa-let-7c-3p | -1.49246 | 1.18E-10 | 4.95E-09 |
| hsa-miR-130b-3p | 1.202625 | 1.11E-14 | 1.68E-12 | hsa-miR-299-5p | -1.49014 | 5.17E-06 | 6.20E-05 |
| hsa-miR-92b-3p | 1.241905 | 6.67E-07 | 1.03E-05 | hsa-miR-451a | -1.43722 | 0.000205 | 0.001527 |
| hsa-miR-767-3p | 1.25035 | 0.001577 | 0.009031 | hsa-miR-30c-2-3p | -1.43259 | 3.81E-08 | 8.10E-07 |
| hsa-miR-455-5p | 1.251536 | 4.49E-12 | 2.69E-10 | hsa-miR-30e-5p | -1.3962 | 2.05E-13 | 2.02E-11 |
| hsa-miR-6087 | 1.255818 | 5.36E-05 | 0.000491 | hsa-miR-135a-5p | -1.38468 | 0.000394 | 0.002641 |
| hsa-miR-187-3p | 1.28345 | 0.000545 | 0.003558 | hsa-miR-1247-3p | -1.33269 | 0.001187 | 0.007063 |
| hsa-miR-7-5p | 1.288334 | 6.78E-08 | 1.39E-06 | hsa-miR-378 | -1.31866 | 2.63E-06 | 3.35E-05 |
| hsa-miR-18a-5p | 1.299273 | 8.30E-11 | 3.67E-09 | hsa-miR-411-5p | -1.31828 | 6.94E-07 | 1.06E-05 |
| hsa-miR-1293 | 1.302229 | 7.07E-07 | 1.06E-05 | hsa-miR-145-3p | -1.31114 | 1.69E-15 | 3.15E-13 |
| hsa-miR-708-3p | 1.302796 | 1.93E-07 | 3.48E-06 | hsa-miR-499a-5p | -1.25484 | 0.003282 | 0.017244 |
| hsa-miR-424-3p | 1.303998 | 1.45E-11 | 7.18E-10 | hsa-miR-337-3p | -1.2377 | 4.49E-07 | 7.38E-06 |
| hsa-miR-2355-3p | 1.32129 | 7.12E-12 | 3.90E-10 | hsa-miR-101-5p | -1.21432 | 1.28E-12 | 9.73E-11 |
| hsa-miR-205-5p | 1.336694 | 1.13E-05 | 0.000125 | hsa-miR-99a-3p | -1.20293 | 1.44E-10 | 5.76E-09 |
| hsa-miR-106b-3p | 1.376878 | 1.43E-12 | 1.04E-10 | hsa-miR-376c-3p | -1.19965 | 2.27E-06 | 3.00E-05 |
| hsa-miR-93-5p | 1.389188 | 1.85E-08 | 4.37E-07 | hsa-miR-125b-5p | -1.17554 | 1.18E-13 | 1.42E-11 |
| hsa-miR-2355 | 1.407882 | 4.44E-13 | 4.01E-11 | hsa-miR-378a-3p | -1.16434 | 4.26E-05 | 0.000395 |
| hsa-miR-1301-3p | 1.419588 | 1.20E-14 | 1.68E-12 | hsa-miR-29a-3p | -1.14176 | 1.35E-10 | 5.52E-09 |
| hsa-miR-1307-3p | 1.424801 | 1.53E-10 | 5.96E-09 | hsa-miR-338-3p | -1.13165 | 2.63E-06 | 3.35E-05 |
| hsa-miR-708-5p | 1.497446 | 1.20E-08 | 3.01E-07 | hsa-miR-144-5p | -1.12615 | 0.00206 | 0.011397 |
| hsa-miR-503-5p | 1.550759 | 1.77E-10 | 6.74E-09 | hsa-miR-199b-5p | -1.12371 | 3.70E-08 | 8.00E-07 |
| hsa-miR-193b-3p | 1.573951 | 6.83E-12 | 3.90E-10 | hsa-miR-410-3p | -1.1024 | 1.12E-06 | 1.61E-05 |
| hsa-miR-937-3p | 1.579378 | 3.46E-11 | 1.61E-09 | hsa-miR-26a-5p | -1.09758 | 8.91E-13 | 7.48E-11 |
| hsa-miR-183-5p | 1.592249 | 2.85E-10 | 1.02E-08 | hsa-miR-29c-5p | -1.09499 | 1.17E-09 | 3.71E-08 |
| hsa-miR-944 | 1.593617 | 7.57E-08 | 1.49E-06 | hsa-miR-378a-5p | -1.0923 | 3.95E-05 | 0.000368 |
| hsa-miR-21-5p | 1.667208 | 1.45E-17 | 4.04E-15 | hsa-miR-338 | -1.08558 | 5.41E-07 | 8.48E-06 |
| hsa-miR-9-5p | 1.669252 | 0.000121 | 0.000975 | hsa-miR-654-3p | -1.08155 | 1.39E-06 | 1.95E-05 |
| hsa-miR-31-3p | 1.703192 | 1.30E-05 | 0.000139 | hsa-miR-495-3p | -1.06692 | 1.13E-05 | 0.000125 |
| hsa-miR-4652-5p | 2.046597 | 4.82E-10 | 1.62E-08 | hsa-miR-379-5p | -1.05545 | 2.54E-06 | 3.27E-05 |
| hsa-miR-224-5p | 2.24261 | 3.16E-12 | 2.04E-10 | hsa-miR-136-5p | -1.03857 | 6.85E-05 | 0.000599 |
| hsa-miR-455-3p | 2.290247 | 2.90E-17 | 6.95E-15 | hsa-miR-126-5p | -1.0353 | 1.00E-10 | 4.32E-09 |
| hsa-miR-31-5p | 2.320609 | 1.19E-08 | 3.01E-07 | hsa-miR-217-5p | -1.02705 | 0.000239 | 0.001756 |
| hsa-miR-767-5p | 2.385888 | 0.000148 | 0.001142 | hsa-miR-190a-5p | -1.00601 | 1.32E-09 | 4.09E-08 |
| hsa-miR-105-5p | 2.578087 | 3.35E-05 | 0.000323 |  |  |  |  |
| hsa-miR-1269b | 2.664693 | 0.001892 | 0.010581 |  |  |  |  |
| hsa-miR-210-3p | 2.715581 | 2.30E-10 | 8.37E-09 |  |  |  |  |
| hsa-miR-1269a | 3.226377 | 1.71E-06 | 2.37E-05 |  |  |  |  |
| hsa-miR-196b-5p | 3.456611 | 2.21E-19 | 9.29E-17 |  |  |  |  |
| hsa-miR-196a-5p | 4.075018 | 1.34E-19 | 7.51E-17 |  |  |  |  |
|  |  |  |  |  |  |  |  |

**Table S3.** Clinical characteristics of Head and Neck squamous cell carcinoma patients. NA, Not available.

| **Variables** | **Case, n (%)** |
| --- | --- |
| **Age at initial diagnosis** | |
| <60 | 214 (44.5) |
| ≥60 | 267（55.5） |
| **Gender** | |
| Male | 350（72.8） |
| Female | 131（27.2） |
| **T stage** | |
| T1 + T2 | 171（35.6） |
| T3 + T4 | 295（61.3） |
| TX | 11（2.3） |
| NA | 4（0.8） |
| **Lymph node status** | |
| N0 | 222（46.2） |
| N1-3 | 238（49.5） |
| NX | 17（3.5） |
| NA | 4（0.8） |
| **Metastasis** | |
| M0 | 452（94.0） |
| M1 | 5（1.0） |
| MX | 20（4.2） |
| NA | 4（0.8） |
| **Stage** | |
| I+ II | 108（22.5） |
| III+ IV | 360（74.8） |
| NA | 13（2.7） |
| **Histologic grade** | |
| G1+G2 | 339（70.5） |
| G3+G4 | 122（25.4） |
| GX | 16（3.3） |
| NA | 4（0.8） |

**Table S4.** Hub genes ranked by overlapping of the top 10 genes according to 11 ranked methods in cytoHubba.

| **Rank methods in cytoHubba** | | | | | | | | | | |
| --- | --- | --- | --- | --- | --- | --- | --- | --- | --- | --- |
| MCC | Eccentricity | Clustering Coefficient | Stress | Betweenness | Bottleneck | DMNC | Degree | EPC | Closeness | Radiality |
| SKP1 | CRKL | ADCYAP1R1 | VAMP2 | VAMP2 | H2AFX | RNF217 | POLR2F | VAMP2 | H2AFX | AR |
| FBXL3 | GGA3 | NFIX | GNAI2 | POLR2F | VAMP2 | RNF126 | SKP1 | H2AFX | SKP1 | CCNB1 |
| KLHL3 | IGF1 | CNTN5 | AR | H2AFX | SFN | UBA6 | GNG4 | SKP1 | POLR2F | H2AFX |
| FBXO9 | IGF1R | RNF217 | MDM2 | GNAI2 | CCNB1 | UBE2V1 | GNG7 | FZR1 | UBE2I | CDK2 |
| UBE2F | CBL | ARHGAP17 | H2AFX | AR | TCF7L2 | UBE2O | GNB4 | CBL | AR | UBE2I |
| FBXW2 | GRIN2B | EDN3 | POLR2F | SFN | POLR2F | TRIM21 | VAMP2 | CD4 | VAMP2 | SKP1 |
| SPSB1 | CAMK2A | RNF126 | UBE2I | DNAJC5 | CBL | UBOX5 | H2AFX | FBXL3 | CCNB1 | GNAI2 |
| FZR1 | CAMK2G | INHBC | CDK2 | CCNB1 | STAT3 | TRIM9 | CBL | FBXW2 | CDK2 | VAMP2 |
| TRIM21 | GRIN2A | CYSLTR2 | LRP8 | SKP1 | HGF | EFTUD2 | RAB1B | M6PR | GNAI2 | GSK3B |
| UBE2V1 | HGF | MED20 | KAT2A | MDM2 | ADCY7 | SF3B3 | GNAI2 | STAM2 | KAT2A | TCF7L2 |

| ID | adj.P.Val | P.Value | t | B | logFC | Gene.symbol | Gene.title |  |  |  |
| --- | --- | --- | --- | --- | --- | --- | --- | --- | --- | --- |
| 8180312 | 4.79E-02 | 4.10E-03 | -3.01171 | -2.289765 | -0.3997156 | SKP1 | S-phase kinase-associated protein 1 | | | |
| 8140556 | 5.63E-02 | 5.14E-03 | -2.92944 | -2.494521 | -0.4708554 | HGF | hepatocyte growth factor | | |  |
| 7952179 | 1.53E-01 | 2.41E-02 | 2.32843 | -3.863151 | 0.2482668 | H2AFX | H2A histone family member X | | |  |
| 8012376 | 1.71E-01 | 2.88E-02 | -2.25245 | -4.018879 | -0.3466127 | VAMP2 | vesicle associated membrane protein 2 | | | |
| 7944493 | 2.21E-01 | 4.40E-02 | 2.067144 | -4.380973 | 0.3030783 | CBL | Cbl proto-oncogene | | |  |
| 7992205 | 3.65E-01 | 1.07E-02 | 1.640589 | -5.11317 | 0.1292226 | UBE2I | ubiquitin conjugating enzyme E2 I | | | |
| 8079964 | 5.98E-01 | 3.31E-01 | 0.982307 | -5.939313 | 0.0528379 | GNAI2 | G protein subunit alpha i2 | | |  |
| 8072979 | 9.10E-01 | 8.21E-01 | 0.228042 | -6.388069 | 0.0290953 | POLR2F | RNA polymerase II subunit F | | |  |

Table S5 The result was validated in external cohorts from the NCI (National Cancer Institute) cohort (GSE10751).
